# Safety Warning about Laparoscopic Power Morcellation in Hysterectomy: A Cost-Effectiveness Analysis of National Impact

**DOI:** 10.1089/whr.2021.0101

**Published:** 2022-03-28

**Authors:** Xiao Xu, Vrunda B. Desai, Peter E. Schwartz, Cary P. Gross, Haiqun Lin, Maria J. Schymura, Jason D. Wright

**Affiliations:** ^1^ Department of Obstetrics, Gynecology and Reproductive Sciences, Yale School of Medicine, New Haven, Connecticut, USA.; ^2^ Yale Cancer Outcomes, Public Policy and Effectiveness Research (COPPER) Center, Yale School of Medicine, New Haven, Connecticut, USA.; ^3^ CooperSurgical, Inc., Trumbull, Connecticut, USA.; ^4^ Department of Internal Medicine, Yale School of Medicine, New Haven, Connecticut, USA.; ^5^ Division of Nursing Science, Rutgers University School of Nursing, Newark, New Jersey, USA.; ^6^ Department of Biostatistics and Epidemiology, Rutgers School of Public Health, Newark, New Jersey, USA.; ^7^ New York State Cancer Registry, New York State Department of Health, Albany, New York, USA.; ^8^ Department of Obstetrics and Gynecology, Columbia University College of Physicians and Surgeons, New York, New York, USA.

**Keywords:** laparoscopic power morcellation, hysterectomy, occult uterine cancer, complications, cost-effectiveness

## Abstract

**Background:**

Following a 2014 safety warning (that laparoscopic power morcellation may increase tumor dissemination if patients have occult uterine cancer), hysterectomy practice shifted from laparoscopic to abdominal approach. This avoided morcellating occult cancer, but increased perioperative complications. To inform the national impact of this practice change, we examined the cost-effectiveness of hysterectomy practice in the postwarning period, in comparison to counterfactual hysterectomy practice had there been no morcellation warning.

**Materials and Methods:**

We constructed a decision tree model to simulate relevant outcomes over the lifetime of patients in the national population undergoing hysterectomy for presumed benign indications. The model accounted for both hysterectomy- and occult cancer-related outcomes. Probability-, cost-, and utility weight-related input parameters were derived from analysis of the State Inpatient Databases, State Ambulatory Surgery and Services Databases, data from the New York Statewide Planning and Research Cooperative System and New York State Cancer Registry, and published literature.

**Results:**

With an estimated national sample of 353,567 adult women, base case analysis showed that changes in hysterectomy practice after the morcellation warning led to a net gain of 867.15 quality-adjusted life years (QALYs), but an increase of $19.54 million in costs (incremental cost-effectiveness ratio = $22,537/QALY). In probabilistic sensitivity analysis, the practice changes were cost-effective in 54.0% of the simulations when evaluated at a threshold of $50,000/QALY, which increased to 70.9% when evaluated at a threshold of $200,000/QALY.

**Conclusion:**

Hysterectomy practice changes induced by the morcellation warning are expected to be cost-effective, but uncertainty in parameter values may affect the cost-effectiveness results.

## Introduction

Hysterectomy (surgical removal of the uterus) is one of the most common gynecologic procedures. Patient safety in hysterectomy is of vital importance given the large number of women undergoing this procedure—over 600,000 women undergo hysterectomy each year in the United States.^1^ Most women undergo hysterectomy for benign indications, such as uterine fibroids, menstrual disorders, and endometriosis.^2^

For women with benign indications, power morcellation (a process that uses a rapidly rotating cylindrical blade to cut and extract tissues) may be used to facilitate the removal of uterus through the small incisions at the time of laparoscopic hysterectomy, especially when large uteri are involved or when the cervix is preserved. By the end of 2013, 59.7% of hysterectomies in the United States were performed laparoscopically and 13.7% of laparoscopic hysterectomies were facilitated by power morcellation.^3^

However, in 2014, the U.S. Food and Drug Administration issued a safety warning cautioning that uncontained power morcellation (hereinafter referred to as morcellation for short) may disseminate cancer cells and impair patients' survival if they have undiagnosed uterine cancer.^4,5^ Although the safety warning centered around patients with occult leiomyosarcoma, which often mimics the appearance of benign fibroids, it broadly affected hysterectomy practice for all benign indications.^6,7^

Minimally invasive laparoscopic hysterectomy helps reduce surgical morbidity and improve patient recovery, compared to the conventional abdominal approach.^8–10^ Yet, the morcellation warning prompted many providers to revert to abdominal hysterectomy to avoid use of power morcellation,^6,7,11^ raising questions about the tradeoff between accidental morcellation of cancerous tissues in laparoscopic hysterectomy and increased risk of surgical morbidity associated with abdominal hysterectomy.^10^ The net impact of these practice changes on the national population remains unknown.

Although several studies have modeled hysterectomy- and occult cancer-related outcomes, they relied on hypothetical patient cohorts (*e.g.*, 100,000 premenopausal women) and assumed that either all patients underwent laparoscopic hysterectomy or all patients underwent abdominal hysterectomy, rather than accounting for the shift in distribution of hysterectomy route in real-world practice.^12–15^

Moreover, these studies focused on patients who underwent hysterectomy for presumed fibroids,^12–14^ despite the fact that the morcellation warning induced widespread change in hysterectomy practice for a broad range of indications.^6,7^ Their modeling of cancer dissemination was also limited to leiomyosarcoma,^12–14^ while other histologic types such as endometrial carcinoma and other sarcomas actually account for a larger share (84%) of occult uterine cancers and morcellation may adversely affect their prognosis as well.^10,16,17^ Thus, findings from prior studies cannot inform the national impact of morcellation warning.

Our study aimed to fill in this gap by evaluating the cost-effectiveness of hysterectomy practice in the national population after the morcellation warning, in comparison to a counterfactual scenario had there been no morcellation warning. We used population-based data on hysterectomy practice changes, accounted for distribution of patient age, and considered the impact of morcellation on both occult endometrial carcinoma and occult uterine sarcoma.

## Materials and Methods

### Overall design

**FIG. 1.**
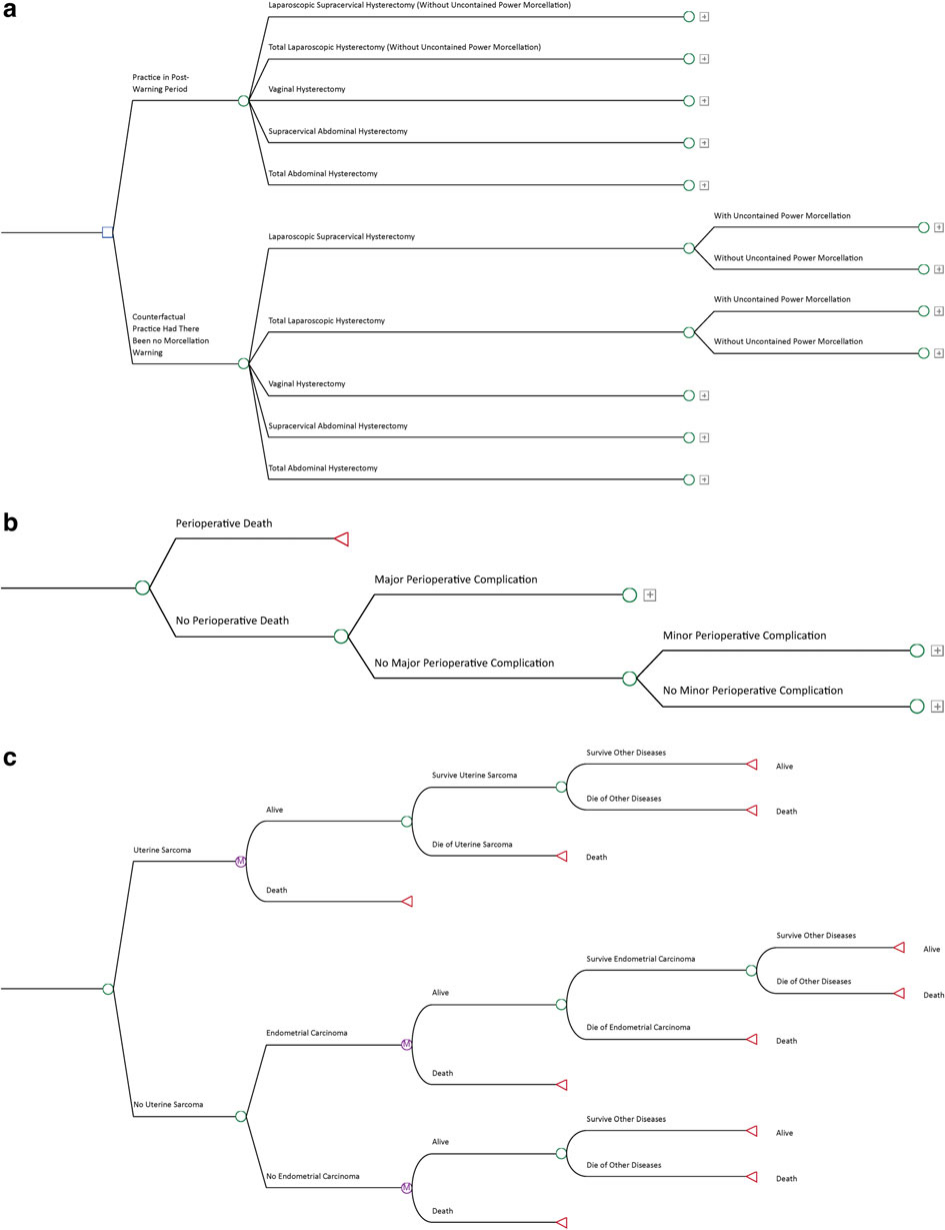
Decision tree model. **(a)** Decision node and hysterectomy route- and morcellation-related health states. **(b)** Subtree reflecting perioperative outcome-related health states. **(c)** Subtree reflecting occult cancer- and survival-related health states. Survival over time in the decision tree model was operationalized using a Markov chain with monthly cycles.

We constructed a decision tree model capturing the relevant outcomes over the lifetime of a patient undergoing hysterectomy for presumed benign indications (Fig. 1a–c). Applying this model to the national population, we compared expected costs and expected quality-adjusted life years (QALYs) between two scenarios: (1) actual hysterectomy practice in the postwarning period, and (2) counterfactual hysterectomy practice had there been no morcellation warning.

Input parameters for this model were derived from three sources: (1) combined data from the State Inpatient Database (SID), State Ambulatory Surgery and Services Database (SASD), and New York Statewide Planning and Research Cooperative System (SPARCS), which provided estimated distribution of hysterectomy route, distribution of patient age, and hysterectomy-related costs and mortality/morbidities; (2) linked SPARCS and New York State Cancer Registry (NYSCR) data, which provided estimated impact of morcellation on mortality risk of occult uterine cancer; and (3) published literature, which provided estimates for all other parameters such as utility weight, productivity loss, and cost of cancer care.

**FIG. 2.**
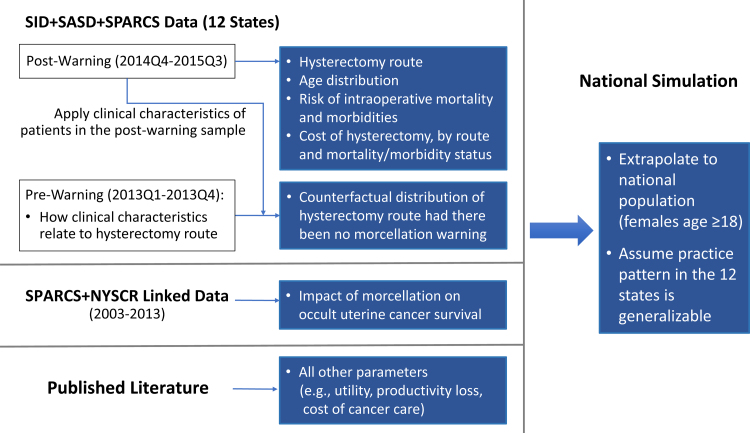
Data management flow diagram. NYSCR, New York State Cancer Registry; SASD, State Ambulatory Surgery and Services Database; SID, State Inpatient Database; SPARCS, Statewide Planning and Research Cooperative System.

Please see Figure 2 for a summary of the data sources used to derive each category of the input parameters in our analysis. This study was approved by the Yale University Institutional Review Board.

### Data

#### Postwarning sample

We obtained SID and SASD data from 11 states in the Agency for Healthcare Research and Quality Healthcare Cost and Utilization Project: Florida, Iowa, Kentucky, Michigan, Minnesota, Nebraska, New Jersey, North Carolina, Oregon, Vermont, and Wisconsin.^18,19^ We additionally acquired inpatient and outpatient discharge data from the New York State SPARCS database.^20.21^

These states were selected because their databases contain admitting diagnosis for both inpatient and outpatient encounters in our study period, which enabled identification of patients who underwent hysterectomy for presumed benign indications. Together, these data provided comprehensive measures of patient clinical information and hospital charges for all surgeries at civilian hospitals in the 12 states, regardless of payer.

Our postwarning sample included women aged ≥18 years in the SID/SASD/SPARCS databases who underwent a hysterectomy for presumed benign indications from 2014Q4 to 2015Q3. We chose this time-period to avoid transitions in practice when the morcellation warning was initially released (in April 2014) and potential confounding effect on measuring practice changes when the International Classification of Diseases (ICD) coding system switched from ICD-9 to ICD-10 after 2015Q3. Hysterectomies were identified using ICD-9 procedure codes and current procedural terminology (CPT) codes.

Patients with presumed benign indications were identified by limiting to women who had an admitting diagnosis of benign gynecologic condition without elevated risk for cancer (*e.g.*, uterine fibroid, endometriosis, genital prolapse) and excluding women who underwent a radical hysterectomy, pelvic evisceration, radiation therapy, chemotherapy, or biopsy/resection procedure or intraoperative pathology consultation typically performed for cancer diagnosis or treatment.

Patients with a discharge diagnosis indicating postmenopausal bleeding or personal history of malignancy were also excluded, as these conditions indicate preoperative elevated risk/evidence for cancer. To reflect a routine gynecologic patient population, we further excluded women who were admitted from the emergency department, were transferred in, or had obstetric conditions.

#### Prewarning sample

We used the SID/SASD/SPARCS data from calendar year 2013 (prewarning period) to facilitate the estimation of counterfactual distribution of hysterectomy route had there been no morcellation warning. The prewarning sample used the same eligibility criteria as the postwarning sample. We selected 2013 as the prewarning period because morcellation warning was released in 2014 and prior research showed that use of power morcellation peaked in 2013.^3,22^

#### Survival sample

We used data on patients with occult uterine cancer from a prior study^17^ to estimate their probability of survival over the lifetime. Inclusion/exclusion criteria of this survival sample are detailed elsewhere.^17^ In brief, this involved women aged ≥18 years with occult endometrial carcinoma or occult uterine sarcoma who underwent a hysterectomy for presumed benign indications from October 1, 2003 to December 31, 2013 in the SPARCS database with linked information from the NYSCR regarding tumor characteristics and mortality.

#### National sample

The 12 study states accounted for 29.89% of the U.S. population of women aged ≥18 years.^23,24^ Therefore, to simulate a national sample, we multiplied the number of women undergoing hysterectomy for presumed benign indications in the above-described postwarning sample by a factor of 1/29.89%. This assumed that the distributions of patient age and practice patterns in the 12 states were generalizable nationwide. A similar approach has been used in prior research.^25^

### Measures

#### Hysterectomy route

We determined hysterectomy route in SID/SASD/SPARCS data based on ICD-9 and CPT procedure codes. Surgical route was classified into the following categories: laparoscopic supracervical hysterectomy (LSH), total laparoscopic hysterectomy (TLH, including laparoscopically assisted vaginal hysterectomy), vaginal hysterectomy, supracervical abdominal hysterectomy (SAH), and total abdominal hysterectomy (TAH).

#### Hysterectomy-related mortality/morbidities

We categorized hysterectomy-related perioperative outcomes in SID/SASD/SPARCS data as in-hospital mortality, major complication, minor complication, or no complication. In-hospital mortality was determined based on the patients' disposition status at the time of discharge. Complications were identified using diagnosis/procedure codes following prior research.^22,26,27^ Major complications included acute myocardial infarction, acute kidney failure, acute pulmonary edema/congestion, operative injury requiring repair, blood transfusion, and other severe morbidities. Minor complications included urinary tract infection, operative wound disruption, hematoma/seroma, electrolyte disturbances, nausea/vomiting, and other mild morbidities.

#### Hysterectomy cost

SID/SASD/SPARCS data from 9 of the 12 study states provided information on hospital facility charges for the entire hospital stay. Charges were converted to costs using hospital-year specific cost-to-charge ratios.^28^ To more accurately reflect the cost-to-charge relationship for patients receiving care for different conditions, the hospital-wide cost-to-charge ratio was refined by a diagnosis-related group (DRG)-specific adjustment factor for inpatient procedures and Clinical Classification Software (CCS) category (based on principal diagnosis code)-specific adjustment factor for outpatient procedures.^28^

Physician fees were estimated as a proportion of hospital facility costs using a validated algorithm based on DRG-specific professional fee ratios for inpatient procedures and CCS category-specific professional fee ratios for outpatient procedures.^29^ Hysterectomy cost included the sum of hospital facility costs and physician fees.

#### Clinical risk factors

We measured patients' age and used diagnosis/procedure codes to categorize their surgical indication (*e.g.*, uterine fibroid, endometriosis, genital prolapse, urinary incontinence, and menopausal disorders), smoking status, comorbidities, and concomitant procedures in SID/SASD/SPARCS data. Comorbidities were measured using the validated algorithm of Elixhauser index and included 29 conditions such as hypertension, diabetes, and obesity.^30,31^ Concomitant procedures were categorized as abdominopelvic procedure (yes/no) and other procedure (yes/no).

#### Uterine cancer-specific survival

For patients with occult uterine cancer in the survival sample, we measured the time (in months) from date of diagnosis to date of death (if patient died of uterine cancer) or the end of follow-up (if patient was alive).^17^ For patients died of other causes, we used their date of death as the date of censoring.

#### Utility weight

Utility weight reflects health-related quality of life associated with a given health state with values ranging from 0 (death) to 1 (perfect health). Utility weights related to the different hysterectomy routes, hysterectomy-related morbidities, and uterine cancer were obtained from the published literature.^12–15,32–46^

#### Other parameters

Values of all other parameters were derived from the published literature. These included the proportion of laparoscopic hysterectomies using morcellation (had there been no morcellation warning), age-specific prevalence of occult endometrial carcinoma and uterine sarcoma, productivity loss associated with hysterectomy and uterine cancer, cost of uterine cancer care, and age-specific risk of mortality from causes other than uterine cancer.^12,15,16,25,47–63^

### Statistical analysis performed to derive input parameters for the decision tree model

#### Estimate counterfactual hysterectomy route

Using data from the prewarning sample, we performed a multinomial logistic regression to examine patients' likelihood of undergoing different hysterectomy route as a function of their clinical characteristics (age, surgical indication, smoking status, comorbidities, and concomitant procedures). Using coefficient estimates from this regression and applying characteristics of patients in the postwarning sample, we predicted the distribution of hysterectomy route in the postwarning period had there been no morcellation warning.

#### Estimate hysterectomy-related morbidity risk

Using data from the postwarning sample, we performed a multinomial logistic regression to examine patients' perioperative outcomes (major complication, minor complication, or no complication) as a function of hysterectomy route, while adjusting for patients' clinical characteristics. Using coefficient estimates from this regression and mean characteristics of patients in the postwarning sample, we estimated the expected risk of major complications and minor complications by hysterectomy route.

We excluded patients with in-hospital mortality from this regression (due to low frequency as an outcome variable) but considered observed risk of in-hospital mortality, along with information in the literature,^15^ to assign mortality risk for each route of hysterectomy.

#### Estimate cost of hysterectomy and related morbidities

Using data from the postwarning sample, we performed a generalized linear regression (with log link and gamma distribution) to examine cost of hysterectomy. The regression included hysterectomy route and indicators of in-hospital mortality, major complication, and minor complication as explanatory variables, while adjusting for patients' clinical characteristics. Using coefficient estimates from this regression and mean characteristics of patients in the postwarning sample, we estimated the expected cost of hysterectomy by surgical route and expected cost of in-hospital mortality, major complication, and minor complication, respectively.

#### Estimate uterine cancer mortality risk associated with morcellation

Using data from the survival sample, we estimated a Weibull survival function for women with occult endometrial carcinoma and occult uterine sarcoma, respectively, undergoing hysterectomy (more detail in Supplementary Appendix SA1). Based on these survival functions and mean characteristics of patients in the postwarning sample, we predicted the probability of survival over time for patients who underwent morcellation and patients who did not undergo morcellation.

### Estimation of the decision tree model

As outlined in Figure 1a–c, we constructed a decision tree model to simulate the lifetime outcome of patients undergoing hysterectomy for presumed benign indications under two scenarios: (1) actual hysterectomy practice in the postwarning period, and (2) counterfactual hysterectomy practice had there been no morcellation warning. The model accounted for the probability, utility weight, and cost associated with the following health states: hysterectomy route, morcellation use, perioperative mortality/morbidities, presence of occult uterine cancer, and subsequent survival over the lifetime. The analysis was conducted from a societal perspective and included both medical costs and patients' productivity loss. Please see Table 1 for a complete list of all input parameters and their values used in the model.

**Table 1.** Input Parameters Used in the Decision Tree Model

Parameter	Base value	95% CI or range^a^	Distribution	References
Probability^b^				
Distribution of hysterectomy route (postwaring)				
TAH	20.58%	—	—	Authors' analysis of SID/SASD/SPARCS data
SAH	6.50%	—	—	
VH	15.29%	—	—	
TLH	51.50%	—	—	
LSH	6.12%	—	—	
Distribution of counterfactual hysterectomy route (had there been no morcellation warning)				
TAH	100% minus the sum of other hysterectomy routes			—
SAH	5.93%	(5.81 to 6.09)	Normal	Authors' analysis of SID/SASD/SPARCS data
VH	15.50%	(15.32 to 15.7)	Normal	
TLH	46.46%	(46.17 to 46.71)	Normal	
LSH	14.01%	(13.79 to 14.22)	Normal	
Proportion of TLH using uncontained power morcellation (postwarning)	0%	—	—	Authors' assumption
Proportion of LSH using uncontained power morcellation (postwarning)	0%	—	—	Authors' assumption
Proportion of TLH using uncontained power morcellation (had there been no morcellation warning)	7.65%	(6.49 to 16.80)	Beta	^56–61^
Proportion of LSH using uncontained power morcellation (had there been no morcellation warning)	75%	(60 to 100)	Beta	^48,49,52,56,62,63^
Probability of perioperative death				
Abdominal hysterectomy	0.02%	(0 to 0.07)	Beta	^15^ and Authors' analysis of SID/SASD/SPARCS data
Vaginal hysterectomy	Same as laparoscopic hysterectomy			Authors' assumption
Laparoscopic hysterectomy	0.01%	(0 to 0.04)	Beta	^15^
Probability of major perioperative complication				
TAH	14.62%	(14.09 to 15.16)	Normal	Authors' analysis of SID/SASD/SPARCS data
SAH	13.16%	(12.35 to 13.97)	Normal	
VH	5.38%	(4.99 to 5.77)	Normal	
TLH	4.21%	(4.04 to 4.39)	Normal	
LSH	3.17%	(2.75 to 3.6)	Normal	
Probability of minor perioperative complication				
TAH	3.92%	(3.64 to 4.2)	Normal	Authors' analysis of SID/SASD/SPARCS data
SAH	3.28%	(2.88 to 3.69)	Normal	
VH	2.03%	(1.79 to 2.27)	Normal	
TLH	1.48%	(1.38 to 1.59)	Normal	
LSH	1.39%	(1.12 to 1.67)	Normal	
Probability of having occult endometrial carcinoma, by age group				
18–29	0.10%	(0.02 to 0.29)	Normal	^16^
30–34	0.11%	(0.04 to 0.18)	Normal	^16^
35–39	0.12%	(0.08 to 0.17)	Normal	^16^
40–44	0.16%	(0.12 to 0.19)	Normal	^16^
45–49	0.28%	(0.23 to 0.32)	Normal	^16^
50–54	0.69%	(0.60 to 0.78)	Normal	^16^
55–59	1.66%	(1.45 to 1.87)	Normal	^16^
60–64	2.47%	(2.17 to 2.76)	Normal	^16^
65–69	2.72%	(2.38 to 3.06)	Normal	^16^
70–74	2.88%	(2.46 to 3.30)	Normal	^16^
≥75	3.93%	(3.47 to 4.38)	Normal	^16^
Probability of having occult uterine sarcoma, by age group				
18–29	0%	—	—	^16^
30–34	0.05%	(0.01 to 0.12)	Normal	^16^
35–39	0.04%	(0.02 to 0.07)	Normal	^16^
40–44	0.11%	(0.08 to 0.13)	Normal	^16^
45–49	0.14%	(0.11 to 0.17)	Normal	^16^
50–54	0.35%	(0.29 to 0.41)	Normal	^16^
55–59	0.55%	(0.43 to 0.67)	Normal	^16^
60–64	0.53%	(0.40 to 0.67)	Normal	^16^
65–69	0.40%	(0.26 to 0.53)	Normal	^16^
70–74	0.26%	(0.13 to 0.39)	Normal	^16^
≥75	0.50%	(0.34 to 0.67)	Normal	^16^
Weibull survival function for occult endometrial carcinoma				
Scale factor associated with uncontained power morcellation	6.05	(4.89 to 7.21)	Normal	Authors' analysis of SPARCS/NYSCR data
Incremental effect of supracervical hysterectomy (without uncontained power morcellation) on scale factor	1.02	(−0.27 to 2.32)	Normal	
Incremental effect of total hysterectomy (without uncontained power morcellation) on scale factor	1.11	(−0.07 to 2.29)	Normal	
Shape parameter	0.82	(0.71 to 0.97)	Normal	
Weibull survival function for occult uterine sarcoma				
Scale factor associated with uncontained power morcellation	4.41	(3.69 to 5.15)	Normal	Authors' analysis of SPARCS/NYSCR data
Incremental effect of supracervical hysterectomy (without uncontained power morcellation) on scale factor	0.78	(−0.04 to 1.61)	Normal	
Incremental effect of total hysterectomy (without uncontained power morcellation) on scale factor	1.02	(0.24 to 1.82)	Normal	
Shape parameter	1.12	(0.95 to 1.32)	Normal	
Utility				
Laparoscopic hysterectomy	0.897	(0.848 to 1)	Beta	^13,15,32^
Vaginal hysterectomy	Same as laparoscopic hysterectomy			Authors' assumption
Abdominal hysterectomy	0.892	(0.72 to 1)	Beta	^13,15,32,33^
Perioperative death	0	—	—	Authors' assumption
Perioperative major complication	0.48	(0.38 to 0.835)	Beta	^13,32–35,45^
Perioperative minor complication	0.61	(0.43 to 0.917)	Beta	^13,32,36,45^
Endometrial carcinoma				
Initial/continuing phase of care^c^	0.83	(0.68 to 0.95)	Beta	^15,37–43,46^
End of life phase,^d^ if died of uterine cancer	0.52	(0.03 to 0.66)	Beta	^12,15,38^
End of life phase,^d^ if died of other causes	Same as initial/continuing phase			Authors' assumption
Uterine sarcoma				
Initial/continuing phase of care^c^	0.67	(0.30 to 0.91)	Beta	^12–15,44^
End of life phase,^d^ if died of uterine cancer	0.52	(0.03 to 0.66)	Beta	^12,15^
End of life phase,^d^ if died of other causes	Same as initial/continuing phase			Authors' assumption
Cost^e^				
Cost of hysterectomy				
TAH	$10,282	($10,216 to $10,348)	Lognormal	Authors' analysis of SID/SASD/SPARCS data
SAH	$9,556	($9,457 to $9,657)	Lognormal	
VH	$8,275	($8,210 to $8,341)	Lognormal	
TLH	$11,641	($11,595 to $11,686)	Lognormal	
LSH	$11,099	($10,978 to $11,222)	Lognormal	
Incremental cost of perioperative death	$18,957	($8,273 to $37,296)	Lognormal	
Incremental cost of perioperative major complication	$4,205	($4,056 to $4,360)	Lognormal	
Incremental cost of perioperative minor complication	$1,471	($1,252 to $1,675)	Lognormal	
Monthly cost of uterine cancer care, <65 years of age				
Initial phase of care^c^	$3,079	($2,801 to $3,359)	Lognormal	^25,50^
Continuing phase of care	$147	($103 to $192)	Lognormal	^25,50^
End of life phase,^d^ if died of uterine cancer	$10,089	($9,723 to $10,456)	Lognormal	^25,50^
End of life phase,^d^ if died of other causes	$425	($59 to $792)	Lognormal	^25,50^
Monthly cost of uterine cancer care, ≥65 years of age				
Initial phase of care^c^	$2,566	($2,288 to $2,846)	Lognormal	^25,50^
Continuing phase of care	$147	($103 to $192)	Lognormal	^25,50^
End of life phase,^d^ if died of uterine cancer	$6,726	($6,360 to $7,093)	Lognormal	^25,50^
End of life phase,^d^ if died of other causes	$425	($59 to $792)	Lognormal	^25,50^
Weekly earnings (productivity loss, if <65 years of age)	$726	($364 to $1,656)	Lognormal	^53^
Recovery time after abdominal hysterectomy (weeks)	5	(4 to 6)	Lognormal	^12,15,55^
Recovery time after vaginal or laparoscopic hysterectomy (weeks)	3	(2 to 4)	Lognormal	^12,15,51^
Monthly cost of uterine cancer-related productivity loss (if <65 years of age)				
Initial phase of care^c^	$203	($192 to $214)	Lognormal	^54^
Continuing phase of care	$83	($72 to $94)	Lognormal	^54^
End of life phase,^d^ if died of uterine cancer	$240	($222 to $260)	Lognormal	^54^
End of life phase,^d^ if died of other causes	Same as continuing phase of care			Authors' assumption

^a^ 95% CI for parameters with a normal or lognormal distribution. Range (*i.e.*, minimum to maximum) for parameters with a beta distribution.

^b^ Other than the listed parameters of probability, the model also accounted for the distribution of patients' age at the time of hysterectomy, which was based on our analysis of patients in the postwarning sample. In addition, age-specific risk of mortality from causes other than uterine cancer was based on the U.S. life table for females in 2015.^47^

^c^ Initial phase of care includes the first 12 months after diagnosis.

^d^ End-of-life phase of care includes the 12 months before death.

^e^ All cost estimates are reported in inflation-adjusted 2015 U.S. dollars.

CI, confidence interval; LSH, laparoscopic supracervical hysterectomy; NYSCR, New York State Cancer Registry; SAH, supracervical abdominal hysterectomy; SASD, State Ambulatory Surgery and Services Database; SID, State Inpatient Database; SPARCS, New York Statewide Planning and Research Cooperative System; TAH, total abdominal hysterectomy; TLH, total laparoscopic hysterectomy (including laparoscopic-assisted vaginal hysterectomy); VH, vaginal hysterectomy.

Each patient in the national sample entered the decision tree model with a randomly assigned age and hysterectomy route based on their distributions, and then accumulated costs and QALYs as she progressed through the various health states over the lifetime. Costs and QALYs occurring in years after the hysterectomy were discounted using a 3% annual rate. All costs were reported in inflation-adjusted 2015 U.S. dollars.

Lifetime costs and QALYs aggregated across all patients in the national sample were compared between the two scenarios: actual hysterectomy practice after the morcellation warning versus counterfactual hysterectomy practice had there been no morcellation warning. Incremental cost-effectiveness ratio (ICER) was calculated as the difference in costs divided by difference in QALYs between these two scenarios.

To account for uncertainty in input parameter values, we specified a distribution for each key input parameter (*e.g.*, beta distribution for utility weights, log-normal distribution for cost parameters) (Table 1), and performed a probabilistic sensitivity analysis using Monte Carlo simulation with 1,000 iterations. In each iteration, the model randomly selected a set of input parameter values (based on their specified distributions) and estimated the expected cost and expected QALY associated with postwarning hysterectomy practice and counterfactual hysterectomy practice had there been no morcellation warning, respectively.

As there is debate regarding the most appropriate benchmark ICER value, we reported the proportion of simulation iterations that were cost-effective at threshold values ranging from $50,000/QALY to $200,000/QALY.^64^ Using results from the 1,000 iterations of the Monte Carlo simulation, we also identified the most influential input parameters^65,66^ (more detail in Supplementary Appendix SA1). Analyses were conducted using SAS 9.4 (SAS Institute, Cary, NC) and TreeAge Pro 2013 (TreeAge Software, LLC, Williamstown, MA).

## Results

### Change in hysterectomy route

In the 12 study states, 108,166 patients and 105,698 patients met eligibility criteria for the prewarning and postwarning sample, respectively (Table 2). Most were 35–54 years of age. Uterine fibroids, menstrual disorders, and endometriosis were the most common indications for hysterectomy.

**Table 2.** Characteristics of Patients in the Prewarning and Postwarning Samples

Characteristics	Prewarning (***N*** = 108,166)		Postwarning (***N*** = 105,698)	
	***N***	%	***N***	%
Age (years)				
18–34	10,241	9.5	10,137	9.6
35–44	41,265	38.1	39,971	37.8
45–54	39,961	36.9	39,145	37.0
55–64	8,529	7.9	8,683	8.2
≥65	8,170	7.6	7,762	7.3
Surgical indication^a^				
Uterine fibroids	61,084	56.5	61,116	57.8
Other benign disorders of the uterus	12,584	11.6	15,033	14.2
Endometriosis	32,934	30.4	33,393	31.6
Pelvic prolapse	21,450	19.8	20,263	19.2
Menstrual disorders	60,993	56.4	57,473	54.4
Menopausal disorders	1,504	1.4	1,558	1.5
Female pelvic inflammatory diseases	29,541	27.3	31,527	29.8
Urinary incontinence	11,450	10.6	10,417	9.9
Disorders of the ovary/fallopian tube	26,905	24.9	30,109	28.5
Noninflammatory disorders of cervix	3,582	3.3	4,349	4.1
Other gynecologic conditions	28,509	26.4	27,526	26.0
Concomitant procedure^a^				
Abdominopelvic	19,752	18.3	16,750	15.8
Other	1,371	1.3	1,595	1.5
Smoking status	17,717	16.4	20,421	19.3
Comorbidities^a^				
Hypertension	23,557	21.8	23,502	22.2
Anemia	15,891	14.7	16,325	15.4
Obesity	11,687	10.8	13,678	12.9
Chronic pulmonary disease	9,842	9.1	10,246	9.7
Hypothyroidism	8,294	7.7	7,878	7.5
Depression	8,577	7.9	8,817	8.3
Diabetes	6,850	6.3	7,137	6.8
No. of other comorbidities				
0	98,366	90.9	95,473	90.3
1	8,633	8.0	8,901	8.4
≥2	1,167	1.1	1,324	1.3

^a^ Conditions/procedures were not mutually exclusive. A patient could have more than one condition/procedure.

Use of LSH, which particularly requires morcellation to remove the corpus uteri while preserving the cervix, decreased substantially after the morcellation warning (Table 3). LSH accounted for 6.1% of the hysterectomies in the postwarning sample, compared to 14.0% of hysterectomies had there been no morcellation warning. Conversely, use of abdominal hysterectomy increased. TAH and SAH accounted for 20.6% and 6.5% of the hysterectomies in the postwarning sample, compared to 18.1% and 5.9%, respectively, had there been no morcellation warning. Meanwhile, use of TLH increased (51.5% in the postwarning sample versus 46.5% had there been no morcellation warning), while use of vaginal hysterectomy remained stable.

**Table 3.** Change in Hysterectomy Route After Power Morcellation Warning

Hysterectomy route	Prewarning sample	Postwarning sample	
	Observed practice	Observed practice	Counterfactual practice (had there been no morcellation warning)^a^
LSH	15,543 (14.4%)	6,473 (6.1%)	14.0% (13.8–14.2)
TLH^b^	49,084 (45.4%)	54,439 (51.5%)	46.5% (46.2–46.7)
Vaginal hysterectomy	17,443 (16.1%)	16,166 (15.3%)	15.5% (15.3–15.7)
SAH	6,528 (6.0%)	6,872 (6.5%)	5.9% (5.8–6.1)
TAH	19,568 (18.1%)	21,748 (20.6%)	18.1% (17.9–18.3)

^a^ Estimated by applying the characteristics of patients in the postwarning sample to coefficient estimates derived from a multivariable regression analysis of hysterectomy route in the prewarning sample. 95% CIs are reported in parentheses.

^b^ Included laparoscopically assisted vaginal hysterectomy.

### National impact on cost and QALY

When extrapolated to a national sample of 353,567 women undergoing hysterectomy for presumed benign indications in 2014Q4–2015Q3 using the decision tree model, base case analysis showed that the practice changes resulted in four additional intraoperative deaths, 1,219 additional patients experiencing a major complication, and 314 additional patients experiencing a minor complication at the time of hysterectomy (Table 4). However, the practice changes prevented morcellating 326 cases of occult endometrial carcinoma and 86 cases of occult uterine sarcoma. These tradeoff effects led to an expected net increase of 867.15 QALYs despite an increase of $19.54 million in societal costs, resulting in an ICER of $22,537/QALY (below the conventional threshold of $50,000/QALY).

**Table 4.** Expected National Impact of the Morcellation Warning, Base Case Analysis

Outcomes	Postwarning practice	Counterfactual practice (had there been no morcellation warning)	Difference
Expected perioperative outcomes			
No. of deaths	49	45	4
No. of patients with a major complication	24,826	23,607	1,219
No. of patients with a minor complication	7,704	7,390	314
Expected cancer outcomes			
No. of patients with occult endometrial carcinoma who underwent uncontained power morcellation	0	326	−326
No. of patients with occult uterine sarcoma who underwent uncontained power morcellation	0	86	−86
Expected total QALY	7,626,699.66	7,625,832.50	867.15
Expected total cost	$4,985,340,993	$4,965,798,124	$19,542,869
Incremental cost-effectiveness ratio		$22,537/QALY	

QALY, quality-adjusted life year.

**FIG. 3.**
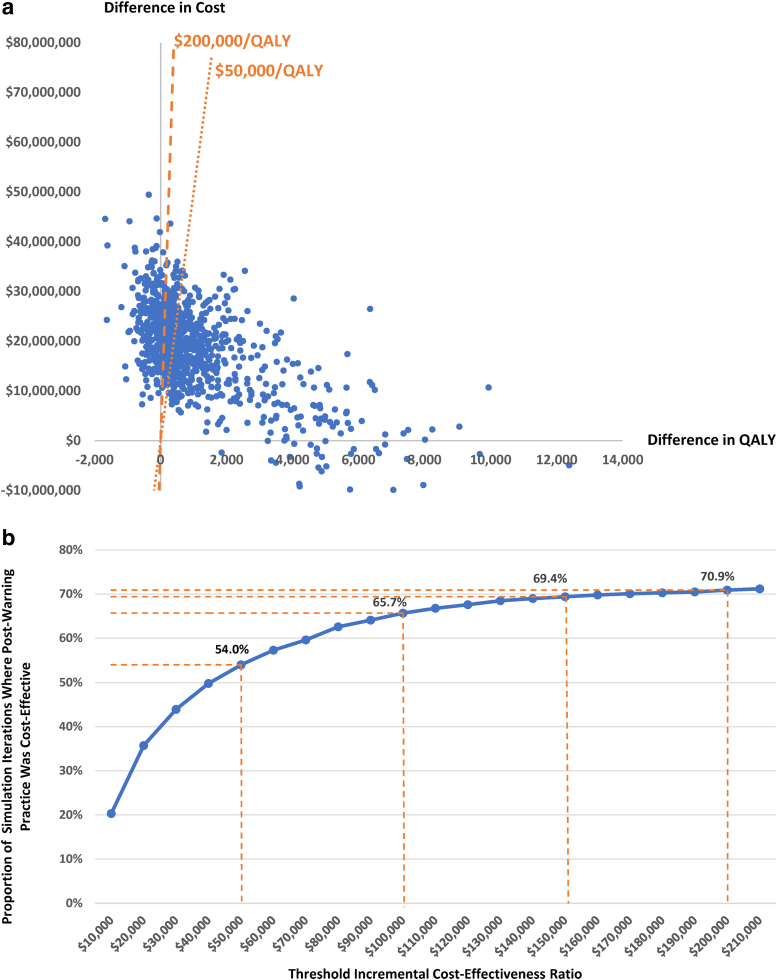
Results from probabilistic sensitivity analysis. **(a)** Incremental cost-effectiveness plane. QALY, quality-adjusted life year. **(b)** Cost-effectiveness acceptability curve.

Figure 3a reports findings from the probabilistic sensitivity analysis via Monte Carlo simulation (*n* = 1,000 iterations) assessing the impact of uncertainty in input parameter values. Each dot in the figure corresponds to result from one iteration of the simulation with regard to difference in expected costs and difference in expected QALYs between postwarning hysterectomy practice and counterfactual hysterectomy practice had there been no morcellation warning. Vertical axis reflects difference in expected costs between the two scenarios, with positive values indicating that expected cost of postwarning hysterectomy practice exceeds expected cost of counterfactual hysterectomy practice had there been no morcellation warning and negative values indicating the opposite.

Horizontal axis reflects difference in expected QALYs between the two scenarios, with positive values indicating that expected QALY of postwarning hysterectomy practice exceeds expected QALY of counterfactual hysterectomy practice had there been no morcellation warning and negative values indicating the opposite. The dotted line corresponds to a threshold value of $50,000/QALY for ICER and the dashed line corresponds to a threshold value of $200,000/QALY for ICER. Dots located to the southeast of these lines are considered cost-effective under these thresholds.

Figure 3b summarizes the proportion of the 1,000 iterations of simulation where postwarning hysterectomy practice is cost-effective, compared to counterfactual hysterectomy practice had there been no morcellation warning, at various threshold values of ICER. Compared to hysterectomy practice without morcellation warning, hysterectomy practice in the postwarning period was cost-effective in 54.0% of the simulations when evaluated at a threshold of $50,000/QALY, which increased to 70.9% when evaluated at a threshold of $200,000/QALY (Fig. 3b).

Input parameters that influenced the simulation results the most included prevalence of occult endometrial carcinoma and uterine sarcoma, impact of morcellation on occult cancer-related mortality, recovery time after hysterectomy, utility weight of abdominal hysterectomy, and proportion of LSH that used uncontained power morcellation had there been no morcellation warning (Supplementary Appendix SA2).

## Discussion

Hysterectomy practice changed in response to the morcellation warning, leading to an increase in hysterectomy-related mortality/morbidity, but a decrease in morcellation of occult cancers. These practice changes are expected to generate a net gain in QALYs and to be cost-effective in base case analysis. However, there remains uncertainty in some parameter values that could affect the cost-effectiveness results.

Although power morcellation facilitates minimally invasive surgery and helps reduce perioperative mortality/morbidity at the time of hysterectomy, it can disseminate occult cancers and adversely affect survival. Prior research suggests cost-effectiveness profiles favoring laparoscopic hysterectomy among younger patients, but favoring abdominal hysterectomy among older patients, since the risk of occult uterine cancer increases with age.^13–15^ By accounting for heterogeneity of patient age in the national population and using data on actual changes in hysterectomy practice, our study extends this literature to address a different question—what is the overall health and financial impact of the morcellation warning at the national level.

After accounting for both hysterectomy- and occult uterine cancer-related effects, we showed that at the national level, hysterectomy practice change induced by the morcellation warning was associated with a net gain in QALYs and was cost-effective in base case analysis. This relieves concerns that the morcellation warning might adversely affect population health by increasing hysterectomy-related surgical complications.

Our finding on changes in hysterectomy route is consistent with the literature. Although research reported decreased use of laparoscopic hysterectomy after the morcellation warning,^7,67^ closer examination showed that the decrease mainly occurred among LSHs, while use of TLH continued to rise.^11,68^ This is not surprising because uterine specimen often can be removed vaginally either intact or after manual morcellation in TLH (without having to undergo power morcellation). As providers continue adapting their practices (*e.g.*, switching from LSH to TLH), in the long run the impact of the morcellation warning on choice of abdominal versus laparoscopic hysterectomy is likely smaller than estimated in our study.

Indeed, a recent study demonstrated that by end of 2016, use of laparoscopic hysterectomy had returned to its projected level had there been no morcellation warning.^68^ Likewise, another study reported that the initial increase in use of abdominal hysterectomy was transient and use of abdominal hysterectomy began decreasing 1 year after the morcellation warning.^69^ Although the latter study^69^ did not adjust for changes in patient case-mix over time and hence the trends in abdominal hysterectomy are yet to be validated, our study likely provides a conservative estimate for the cost-effectiveness of the morcellation warning.

Nevertheless, continued research is needed to monitor the safety of manual morcellation and contained power morcellation that have been proposed to replace uncontained power morcellation. Manual morcellation may also pose some risk for disseminating cancer cells, which can have safety implications if power morcellation was largely substituted by TLH with the use of manual morcellation. Unfortunately, empirical evidence about how manual morcellation affects the prognosis of patients with occult uterine cancer remains sparse and inconclusive in the current literature.^10,70^

Likewise, concerns about the safety of contained power morcellation (*e.g.*, perforation of the containment bag, leakage, and injury due to obstructed visual field) also remain.^10^ Due to lack of adequate data on these issues, our simulation of national impact did not account for these factors. Enhancing research in these areas will allow us to evaluate the impact of the morcellation warning more thoroughly.

This study also revealed uncertainty in several input parameters (*e.g.*, prevalence of occult uterine cancer and impact of morcellation on uterine cancer survival) that could considerably affect the cost-effectiveness results. Despite growing research, our understanding of these parameters is still limited and warrants further investigation.^70^

In addition, endometrial carcinoma-related parameters were among the most influential factors identified in our analysis, yet, prior research mostly focused on occult leiomyosarcoma. Although endometrial tissue sampling is readily available and effective in detecting endometrial carcinoma preoperatively, it might have been underutilized such that endometrial carcinoma accounts for 78% of patients with occult uterine cancer undergoing hysterectomy in the prewarning era.^16^ These patients were also subject to an increased risk of tumor dissemination if they underwent power morcellation.^10^ By accounting for these patients in our analysis, our study provided a more comprehensive evaluation of the national impact of the morcellation warning.

Major strengths of this study include our use of population-based data from 12 states across the country (enhancing generalizability of the findings) and comprehensive assessment incorporating both endometrial carcinoma and uterine sarcoma. However, we do recognize several limitations of this study.

First, we relied on administrative data, which lack sufficient detail regarding patients' preoperative evaluations and clinical circumstances. This could limit the accuracy and adequacy in measuring risk factors and surgical outcomes, as well as our ability in identifying patients with presumed benign indications. Hence, we imposed strict sample inclusion/exclusion criteria (*e.g.*, requiring patients to have an admitting diagnosis of clear benign gynecologic condition). As hysterectomy may be performed for less specific indications (*e.g.*, abdominal pain) or secondary to nongynecologic procedures (*e.g.*, gastrointestinal procedures), our analysis may underestimate the national impact.

Second, since there is no database that can provide nationally representative data encompassing both inpatient and outpatient hysterectomies, we used statewide data from 12 states and extrapolated their experience to the entire country. It is likely that the morcellation warning may affect hysterectomy practice differently in these states than elsewhere in the country.

## Conclusions

Hysterectomy practice after the morcellation warning was expected to be cost-effective than a counterfactual scenario had there been no morcellation warning. However, continued effort is needed to improve the quality of scientific evidence around the prevalence of occult uterine cancer at the time of hysterectomy, impact of morcellation on patient survival, and the safety of manual morcellation and contained power morcellation. Enhanced knowledge in these areas can better guide clinical and policy decisions to help improve population health.

## Supplementary Material

Supplemental data

Supplemental data

## Supplementary Material

Supplementary Appendix SA1

Supplementary Appendix SA2

## Abbreviations Used

CCS: Clinical Classification Software
CI: confidence interval
CPT: current procedural terminology
DRG: diagnosis related group
ICD: International Classification of Diseases
ICER: Incremental cost-effectiveness ratio
LSH: laparoscopic supracervical hysterectomy
NYSCR: New York State Cancer Registry
QALYs: quality-adjusted life years
SAH: supracervical abdominal hysterectomy
SASD: State Ambulatory Surgery and Services Database
SID: State Inpatient Database
SPARCS: Statewide Planning and Research Cooperative System
TAH: total abdominal hysterectomy
TLH: total laparoscopic hysterectomy
