# Supplementary material for: Safety Warning about Laparoscopic Power Morcellation in Hysterectomy: A Cost-Effectiveness Analysis of National Impact
Source: Womens Health Rep (New Rochelle). 2022 Mar 28;3(1):369–84. doi: 10.1089/whr.2021.0101 (PMC8994439; doi:10.1089/whr.2021.0101)
Supplement: Supplemental data [file Suppl_AppendixSA1.docx]

**Appendix A.** Additional Technical Detail

Estimation of Weibull Survival Function: To facilitate the prediction of lifetime survival, we re-analyzed data from a prior study (Xu et al. 2019) which contains a sample of patients who had occult uterine cancer and underwent hysterectomy via different surgical routes. This re-analysis of the data was necessary because the prior study (Xu et al. 2019) only reported results from a semi-parametric, cox proportional hazards survival function, which could not predict patients’ likelihood of survival beyond their follow-up period. Since our simulation model requires input parameters about patients’ likelihood of survival over the rest of their lifetime, we re-analyzed the data in Xu et al. (2019) to estimate a parametric Weibull survival function which allowed for prediction of a patient’s likelihood of survival at any given timepoint in life.

Details about the design of the prior study are reported elsewhere (Xu et al. 2019). Briefly, the analysis used linked data from the New York Statewide Planning and Research Cooperative System (SPRACS) and the New York State Cancer Registry to identify adult women with occult uterine cancer who underwent a hysterectomy for presumed benign indications from October 1, 2003-December 31, 2013. Using laparoscopic supracervical hysterectomy (LSH) as a surrogate indicator for uncontained power morcellation (which was the standard technique of tissue removal for LSH prior to the morcellation warning), we compared uterine cancer-specific survival of patients who underwent LSH versus those who underwent supracervical abdominal hysterectomy (SAH) and those who underwent total abdominal hysterectomy (TAH) using a Weibull survival function. A propensity score adjustment method was used to account for differences of patient characteristics across hysterectomy routes. Separate analyses were performed for patients with occult endometrial carcinoma and patients with occult uterine sarcoma. Patients who underwent total laparoscopic hysterectomy (TLH) or vaginal hysterectomy (VH) were excluded from this Weibull regression analysis because of uncertainty in their use of power/manual morcellation. For the purpose of the current study, we assumed that patients who underwent TLH with uncontained power morcellation had the same survival function as patients who underwent LSH and that patients who underwent TLH without power morcellation or underwent VH had the same survival function as patients who underwent TAH.

Probabilistic Sensitivity Analysis: To account for uncertainty in input parameters, we specified a distribution for each key input parameter (e.g., beta distribution for utility weights, log-normal distribution for cost parameters) and performed a probabilistic sensitivity analysis using Monte Carlo simulation with 1,000 iterations. In each iteration, the model randomly selected a set of input parameter values (based on their specified distributions) and estimated the expected cost and expected quality-adjusted life years (QALY) associated with post-warning hysterectomy practice and counterfactual hysterectomy practice had there been no morcellation warning, respectively. Using these expected costs and expected QALYs, we calculated the incremental net monetary benefit (INMB) between post-warning practice and counterfactual practice had there been no morcellation warning, where INMB = (difference in QALY * willingness to pay for each QALY) - difference in cost. We used the conventionally accepted willingness to pay of $50,000 per QALY. After 1,000 iterations of the simulation, we essentially had 1,000 sets of input parameter values along with their corresponding INMB values. Using these data, we conducted a general linear regression with INMB as the outcome variable and values of all input parameters as the explanatory variables. Input parameters with the largest standardized coefficient estimates (which reflected the impact on INMB associated with one standard deviation change in the input parameter value) were considered the most influential parameters (Helton 1993; Iman et al. 1988).

We used INMB, instead of incremental cost-effectiveness ratio (ICER), in this assessment because INMB has a monotonic relationship with costs and QALYs such that higher values of INMB always indicate more preferred results. In contrast, ICER is a ratio and has a non-monotonic relationship with costs and QALYs. For instance, ICER can take negative values when an intervention is more effective and less costly than the comparison intervention (which is a desired scenario) or when an intervention is less effective and costlier than the comparison intervention (which is an undesired scenario).

References:

Helton JC. Uncertainty and sensitivity analysis techniques for use in performance assessment for radioactive waste disposal. Reliability Engineering and System Safety. 1993;42(2): 327-367.

Iman RL, Helton JC. An investigation of uncertainty and sensitivity analysis techniques for computer models. Risk Analysis. 1988;8(1):71-90.

Xu X, Lin H, Wright JD, Gross CP, Boscoe FP, Hutchison LM, Schwartz PE, Desai VB. Association between power morcellation and mortality in women with unexpected uterine cancer undergoing hysterectomy or myomectomy. J Clin Oncol. 2019;37(35):3412-3424.
