# Supplementary material for: Safety Warning about Laparoscopic Power Morcellation in Hysterectomy: A Cost-Effectiveness Analysis of National Impact
Source: Womens Health Rep (New Rochelle). 2022 Mar 28;3(1):369–84. doi: 10.1089/whr.2021.0101 (PMC8994439; doi:10.1089/whr.2021.0101)
Supplement: Supplemental data [file Suppl_AppendixSA2.docx]

**Appendix B**. Most influential input parameters

| **Ranking** | **Input Parameter** | **Standardized Coefficient** |
| --- | --- | --- |
| 1 | Probability of having occult endometrial carcinoma | 0.6956 |
| 2 | Occult endometrial carcinoma: Incremental effect of total hysterectomy (without morcellation) on scale factor of Weibull survival function | 0.1721 |
| 3 | Probability of having occult uterine sarcoma | 0.1618 |
| 4 | Occult endometrial carcinoma: Scale factor of Weibull survival function associated with uncontained power morcellation | -0.0890 |
| 5 | Occult uterine sarcoma: Incremental effect of total hysterectomy (without morcellation) on scale factor of Weibull survival function | 0.0678 |
| 6 | Recovery time after abdominal hysterectomy | -0.0627 |
| 7 | Recovery time after vaginal/laparoscopic hysterectomy | 0.0617 |
| 8 | Occult endometrial carcinoma: Incremental effect of supracervical hysterectomy (without morcellation) on scale factor of Weibull survival function | 0.0510 |
| 9 | Utility weight of abdominal hysterectomy | 0.0470 |
| 10 | Proportion of laparoscopic supracervical hysterectomy using uncontained power morcellation in counterfactual practice (had there been no morcellation warning) | 0.0448 |
